# Effect of large-scale mass drug administration for malaria on mortality and morbidity in Angumu health zone, Ituri, Democratic Republic of Congo

**DOI:** 10.1186/s12936-023-04469-7

**Published:** 2023-02-06

**Authors:** Lise Grout, Yves Katuala Givo, Trish Newport, Tom Adoum Mahamat, Priscillah Gitahi, Jean Jacques Mandagot, Michel Quere, Sophie Wodon, Iza Ciglenecki, Mathieu Bastard, Francis Baelongandi, Louis Tshulo, Herman Jakisa Uluba, Esther Sterk, Etienne Gignoux

**Affiliations:** 1 Epicentre, Geneva, Switzerland; 2 Médecins Sans Frontières, Goma, Democratic Republic of Congo; 3 Médecins Sans Frontières, Geneva, Switzerland; 4 Ministry of Health, Bunia, Democratic Republic of Congo

**Keywords:** Malaria, Mass drug administration (MDA), Prevention, Mortality, Democratic Republic of Congo

## Abstract

**Background:**

Angumu health zone in Ituri, Democratic Republic of Congo, is a highly malaria-endemic area with an overburdened health system and hosting internally displaced persons (IDP). The World Health Organization recommends mass drug administration (MDA) for malaria in complex emergencies. Therefore, three MDA rounds were implemented by Ministry of Public Health and Médecins sans Frontières from September 2020 to January 2021 in four health areas selected for epidemiological (high malaria incidence) and logistic reasons. Reported mortality and morbidity were compared in locations where MDA has been performed and locations where it has not.

**Methods:**

A non-randomized controlled population-based retrospective mortality survey was conducted in March 2021. Two-stage cluster sampling was used in villages; all IDP sites were surveyed with systematic random sampling. The main (mortality rates) and secondary (morbidity) outcomes were estimated and compared between locations where MDA had been conducted and where it had not, using mixed Poisson and binomial regression models respectively.

**Results:**

Data was collected for 2554 households and 15470 individuals, of whom 721 died in the 18-month recall period. The under-five mortality rate (U5MR) decreased in the locations where MDA had been implemented from 2.32 [1.48–3.16] “before” the MDA to 1.10 [0.5–1.71] deaths/10,000 children under 5 years/day “after”, whereas it remained stable from 2.74 [2.08–3.40] to 2.67 [1.84–3.50] deaths/10,000 children/day in the same time periods in locations where MDA had not been implemented. The U5MR and malaria-specific mortality was significantly higher in non-MDA locations after MDA was implemented (aRR = 2.17 [1.36–3.49] and 2.60 [1.56–4.33], respectively, for all-cause and malaria-specific mortality among children  < 5 years). Morbidity (all age and  < 5 years, all cause or malaria-specific) appeared lower in MDA locations 2.5 months after last round: reported malaria-specific morbidity was 14.7% [11–18] and 25.0% [19–31] in villages and IDP sites where MDA had been implemented, while it was 30.4% [27–33] and 49.3% [45–54] in villages and IDP sites with no MDA.

**Conclusions:**

Despite traditional limitations associated with non-randomized controlled retrospective surveys, the documented sharp decrease of under-5 mortality and morbidity shows that MDA has the potential to become an important malaria-control tool in emergency settings. Based on these results, new MDA rounds, along with indoor residual spraying campaigns, have been planned in the health zone in 2022. A set of surveys will be conducted before, during and after these rounds to confirm the effect observed in 2021 and assess its duration.

**Supplementary Information:**

The online version contains supplementary material available at 10.1186/s12936-023-04469-7.

## Background

Since December 2017, the province of Ituri in the Democratic Republic of Congo (DRC) has once again been plunged into violence, causing waves of massive population movements towards the safe villages of the Angumu health zone, on the shore of Lake Albert and left bank of the Kakoy River. In February 2019, the emergency team of Médecins Sans Frontières—Operational Centre Geneva (MSF-OCG) in DRC carried out an assessment of the humanitarian situation in Angumu health zone and estimated that there were around 29,000 displaced people scattered among host families in the villages and in internally displaced people (IDP) camps. The under-five mortality rate (U5MR) among IDPs over a 7-month recall period was above the emergency threshold (3.2 deaths per 10,000 persons per day; 95% CI 2.8–3.6) and the main causes of mortality were malaria (36.6%), anaemia (16.2%) and diarrhoeal diseases (14.9%) [1]. To respond to this medical-humanitarian emergency, MSF-OCG has been running the Angumu project since May 2019.

Because of its climate, and in particular its rainfall, Angumu is extremely exposed to malaria, which remains present throughout the year. It can be considered as holoendemic, as the prevalence is very high and the children under 5 years of age are the ones expressing more severe symptoms, whilst the older may rather be asymptomatic, or with reduced damage, due to adaptive immunity. The years 2019 and 2020 have seen the heaviest rainfall for more than a decade. Faced with this dramatic situation, MSF, in collaboration with Ministry of Health (MoH), has increased the population's access to healthcare by supporting eight health centres and setting up 13 community care sites in IDP camps. In addition, impregnated mosquito nets were distributed. However, despite these malaria control interventions, the incidence of malaria remained very high. In January 2020, a mortality survey carried out by MSF-OCG showed a crude mortality rate (CMR) above the emergency threshold in the villages and in the IDP camps (respectively 1.53 (95%CI 1.23–1.82, design effect 4.0) and 1.17 (IC 95%:1.03–1.32, design effect 1.2) deaths/10,000 population/day). The U5MR was also above the emergency threshold in villages with 3.7 (95% CI 2.61–4.79, design effect 4.4) deaths per 10,000 children per day, and in IDP camps, 3.27 (95% CI 2.67–3.86, design effect 1.1) deaths per 10,000 children per day. The survey showed that malaria was the main cause of death, particularly among children under 5 years old [2].

In this context of a complex humanitarian crisis, population movement, very high mortality caused by malaria, limited access to care and prevention measures, and potentially overcrowded health structures in the context of the COVID-19 pandemic, the use of mass drug administration (MDA) against malaria was justified in accordance with the recommendations of the World Health Organization (WHO) [3]. The implementation of MDA aims to rapidly reduce malaria mortality and morbidity and has an initial short-term impact. Moreover, by reducing the prevalence of the parasite in the community and among vectors, MDA, if it has good coverage and is carried out synchronously, can, in conjunction with preventive and curative actions, have a longer-term impact due to reduction in transmission level [4].

**Fig. 1:**
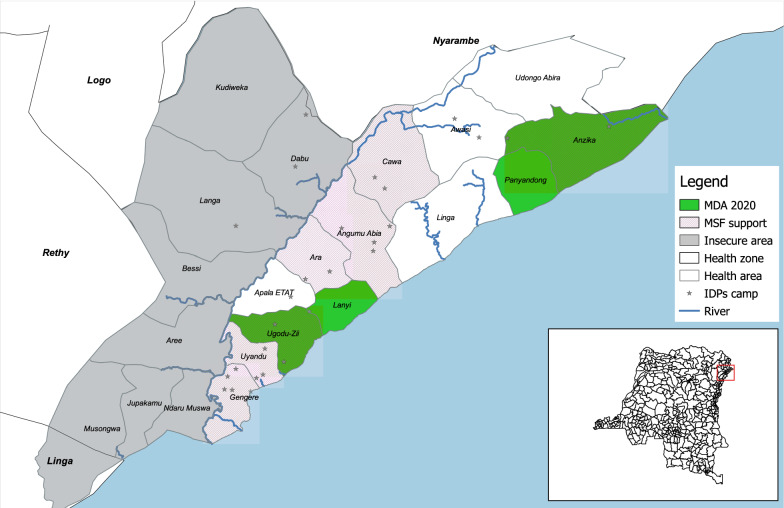
Health areas targeted for the Mass drug administration (MDA) campaigns from September 2020 to January 2021 in Angumu health zone, Ituri province, DRC. *MDA *Mass drug administration; *MSF* Médecins Sans Frontières; *IDP* Internally displaced persons. *ASAQ* 3-day regimen of Artesunate-Amodiaquine. *PIRAMAX* 3-day regimen of Artesunate-Pyronaridine. Note that the health areas are represented on the map as they were in 2020. Since then, Anzika health area has been divided into two health areas (Anzika and Pakala)

MDA campaign was launched in September 2020 by MSF and MoH. The whole population living in the villages and IDP camps of 4 health areas (HA), namely Ugudo-Zii, Lanyi and Panyandong and Anzika (divided into two health areas (Anzika and Pakala) in 2022) (Fig. 1), were targeted by three MDA rounds: 2 rounds of 3-day regimen of artesunate-amodiaquine (ASAQ) in October and November 2020 and 1 round of 3-day regimen of artesunate-pyronaridine (Pyramax^®^) in December 2020. ASAQ is the first-line treatment regimen in DRC; it was, therefore, easy to procure and expected to meet good acceptance and adherence by the population. It had already been used in other MDA campaigns, e.g. by the MoH in DRC in 2018 during the Ebola outbreak and by MSF during the West African Ebola outbreak. Pyramax^®^ has been prequalified by the WHO more recently, is included in the WHO Guideline for the Treatment of Malaria (2022 revision) and considered as having a good safety profile. As ASAQ, besides killing the malaria parasite, it gives a long prophylactic effect. Pyramax^®^ is MSF’s first option in COVID outbreaks due to best safety profile when compared with other treatments and was, therefore, chosen for the third round in a context of COVID19 pandemic where cases could have been under detected due to weak health system. There was no selection in who received which drug. The whole population received ASAQ during the first two rounds and Pyramax^®^ in the third round.

To reduce the risk of COVID-19 transmission, the drugs were distributed door-to-door by 227 teams of 22 community health workers to all people aged 2 months or older and living in the four targeted HAs, selected for epidemiological (high malaria incidence) and logistic reasons. The first dose was taken under direct observation, while the pills for day 2 and 3 were given to the head of the household or the beneficiaries. All people in the targeted areas were eligible for MDA except children under 2 months of age, pregnant women in the first trimester of pregnancy, severely ill people and those who had taken malaria treatment in the 15 days prior to the distribution.

**Fig. 2:**
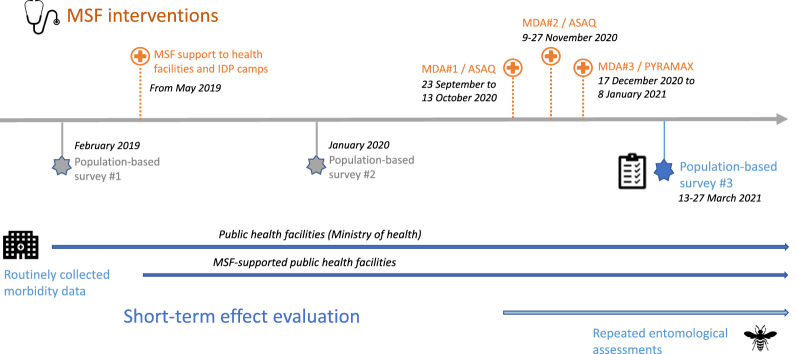
Timeline of malaria interventions and the different components of the assessment, from February 2019 to March 2021, in Angumu health zone, Ituri province, DRC

There was at the time of the survey very little documentation on the use of MDA in “complex emergency setting” as per WHO recommendations. Moreover, this campaign was the first MDA for malaria conducted in high malaria prevalence area outside of an Ebola context, in a context of complex emergency and in the midst of Covid-19 pandemic. To document feasibility of the implementation, the implementational teams collected routine mass campaign indicators, including monitoring of adverse events. To document the potential impact of such MDA campaigns in complex emergency settings, routinely collected malaria morbidity data from the health facilities in areas targeted and not-targeted by MDA were analysed, and a retrospective population-based survey was conducted (Fig. 2). This paper focuses on the survey, for which the main objective was to describe the short-term effect of MDA in Angumu health zone by comparing mortality and morbidity between HAs targeted or not for the MDA, and before and after the MDA took place.

## Methods

A non-randomized controlled population-based retrospective mortality survey was conducted from 13 to 27 March 2021.

### Study setting

There were 20 HAs in the Agumu health zone in 2020, but 7 were not accessible due to insecurity. The survey was conducted in 13 accessible HA in Angumu: the 4 HAs which received MDA (called “MDA locations”), and 9 HAs which had not received MDA (called “non-MDA locations”). The target population for the survey was the entire population of the 13 HAs (estimated at 150,000 people), regardless of their status (resident or IDP). A person was included in the study if s/he lived in the selected household and informed consent was given by the head of the family. A person was excluded from the study if s/he refused to participate or was not located after two attempts to find him/her.

### Outcomes

As children under 5 years of age are at higher risk of malaria-related deaths but the whole population was treated, the main outcome was crude and under-five mortality rates (CMR and U5MR). The secondary outcomes were malaria-specific mortality rate, crude, under-five morbidity and malaria-specific morbidity 2 weeks prior to the survey. MDA coverage for the different rounds was also estimated.

The recall period was chosen to allow comparison of mortality rate before and since the start of MDA activity. Given the seasonal nature of malaria, it is necessary to have the same months of the year in both sub-periods. The recall period therefore started on 2 September 2019 (back to school day) and ended on the day of the survey, which corresponds to 557 days. It was divided into two periods: “before” from 2 September 2019 to 30 September 2020 and “after” from 1st October 2020 to the day of the survey. The “before” period was sub-divided into two equal periods for the calculation of the mortality rates.

### Sample size and sampling strategy

The situation in IDP camps and villages is different in many aspects that could affect the study outcomes (e.g. population density assistance received, access to health care, vulnerabilities). The sample was stratified by location, i.e. villages *versus* IDP camps. All IDP camps were included in the survey and a random systematic sampling was conducted. For the villages, the sampling strategy was further stratified between HAs where MDA was conducted versus those where MDA was not. Two-stage proportional to size random sampling was then conducted in each stratum.

The sample size was calculated to detect a 50% difference in U5MR (from 3 deaths/10,000 children/day to 1.5 deaths/10,000 children/day) between targeted and non-targeted locations “after” the MDA. For a 152 day recall period (“after”), an expected design effect of 2, an average household size of 6.7 and an expected proportion of children under 5 years of age of 20% in selected households, 786 households were needed in each stratum (MDA/non MDA) for the villages and 731 households in each stratum for the IDP camps, respectively; 31 clusters of 26 households were randomly selected in each stratum of the villages, and a sampling interval of 15 households was used in the IDP camps.

### Data collection

All interviewers and supervisors were recruited locally and received theoretical and practical training before conducting the interviews. Selected households (defined as a group of people who live together under the responsibility of one person, and share their meals at least 3 times a week, whether or not they have a family relationship), and were revisited later in the day if the head of the household or an adult was absent, and not replaced if absent at second visit.

Interviewers used a standardized pre-tested questionnaire to collect information on the household, the people living in the household (age, sex, MDA receipt for the 3 rounds, compliance with 3 days treatment for the last round, morbidity and health care use in the 2 weeks prior to the survey) and the people who left the household (date of departure) or died (date of death, cause of death, health care use prior to the death) in the recall period. Data were collected using Kobo Toolbox and the Kobo Collect Application on a tablet, which is a free and open-source software initially developed for humanitarian use by the Harvard Humanitarian Initiative, Harvard TH Chan School of Public Health, and the Brigham and Women’s Hospital [5], and hosted on MSF servers in Belgium.

### Data analysis

Descriptive analysis of the studied population was first conducted, in terms of size and demographic characteristics. Then, the main and secondary outcomes were estimated, taking into account the sample design by applying weights and declaring the design through svy package [6] in R software. These outcomes were compared between locations where MDA was conducted versus locations where it was not (“here” and “there” comparison), and “before” and “after” the MDA was conducted.

The departure date for people who left the household during the recall period was not collected. In calculating mortality rates, these departed individuals counted for half the recall period. Since the explanatory data analysis was split into “before” and “after” MDA, a departure date was randomly assigned during the recall period to all persons who left.

After pooling villages and IDP camps together, a weighted Kaplan–Meier estimates was used to draw survival curves and log-rank test was run to compare the mortality in the two groups (MDA and non-MDA locations). For the explanatory analysis, the hypothesis was that mortality and morbidity would be significantly higher in “non-MDA locations” than in “MDA locations” after the MDA was conducted, adjusting on other factors. A mixed Poisson regression was performed to estimate adjusted mortality rate ratio (aMRR) and a mixed binomial regression to estimate adjusted relative risks (aRR) of being sick in the 2 weeks prior to the survey. Clusters were considered as random effects in both regression models to allow for intra and inter cluster correlation.

R open-source software (v 4.1.2) [7] and Stata^©^ version 16 (Stata-Corp LP, College Station, TX, USA) were used for the statistical analysis.

### Ethical considerations

This study was sponsored by MSF. The authors assume full responsibility for the analyses and interpretation of the data. This study was conducted according to the ethical principles for research on human subjects, described in the Helsinki Declaration, and in accordance with international principles and guidelines for biomedical research involving human subjects, published by the Council for International Organizations of Medical Sciences [8]. The study protocol was approved by the MSF Ethics Review Board and the Kisangani University of DRC.

MDA was provided free of charge. Participation in the study was voluntary and oral informed consent was obtained from the head of the household or another adult in charge.

## Results

From 13 to 27 March 2021, 1603 and 951 households were visited respectively in selected villages and IDP camps, with 9954 and 5440 individuals included, respectively. No household refused to participate to the survey. In the camps, 98.2% of the households were internally displaced, while they were 22% in the villages. The size of the surveyed households was 5.5 in the villages and 5.0 in the camps. The age pyramid was slightly different between camps and villages, but comparable between MDA and non-MDA sites (Additional file 1).

The following sections describe the main results regarding the coverage of the MDA campaigns, the mortality (under 5, crude and malaria specific) and the morbidity (under 5, crude and malaria specific).

### MDA coverage

The coverage of the three rounds of MDA was  > 95% in the IDP camps and  > 90% in the villages (Table 1). It was  > 90% in IDP camps and  > 85% in villages when considering only the vaccination cards.

**Table 1:** Coverage of the different rounds of mass drug administration against malaria in 2020 in Angumu health zone, Ituri province, Democratic Republic of Congo

	Villages			IDP camps		
	% (N)	95% CI	Design effect	% (N)	95%CI	Design effect
ASAQ1						
Documented	85% (3443)	[81–89]	16.7	92.9% (1238)	[91–95]	2.9
Orally reported	6.8% (274)	[4–10]	13.6	3.8% (50)	[2–6]	3.6
* Missing*	*38*			*0*		
Overall coverage	90.5% (3717)	[88–94]	11.8	96.6% (1288)	[95–98]	1.9
ASAQ2						
Documented	85.1% (3448)	[81–89]	15.7	93.1% (1240)	[91–95]	2.9
Orally reported	7% (282)	[4–10]	13.2	3.8% (50)	[2–6]	3.6
* Missing*	*34*			*0*		
Overall coverage	90.9% (3730)	[88–94]	11.8	96.7% (1290)	[95–98]	1.9
PYRAMAX						
Documented	84.7% (34343225)	[80–89]	15.3	93.3% (1243)	[91–96]	3
Orally reported	7.3% (291)	[4–10]	12.6	3.8% (50)	[2–6]	3.6
* Missing*	*33*			*0*		
Overall coverage	90.9% (3724)	[88–94]	11.8	96.7% (1293)	[95–98]	1.9

*95% CI* 95% confidence interval, *IDP* Internally Displaced People, *ASAQ1* 1st round of Artesunate Amodiaquine, *ASAQ2* 2nd round of Artesunate Amodiaquine, *PYRAMAX* Round of Artesunate Pyronaridine

### Mortality

For the whole recall period, 645 deaths were reported, 426 in the “Non-MDA locations” and 229 in the “MDA locations”. The overall CMR was 1.23 [1.05;1.41] deaths/10,000 population/day (deff = 2.74), while the U5MR was 2.23 [1.75;2.71] deaths/10,000 children under 5 years of age/day (deff = 2.11) (Additional file 2).

The CMR was very similar in “non-MDA” and “MDA locations” before the MDA campaign (respectively 1.07 [0.84–1.30] and 0.99 [0.68–1.29] deaths/10,000 population/day) and remained very similar after the MDA campaign started (respectively 0.92 [0.78–1.06] and 0.80 [0.51–1.08] deaths/10,000 population/day) (Table 2).

**Table 2:** Mortality rates (crude, under-five and malaria-specific) in locations where mass drug administration has been implemented (MDA locations) and where it has not (Non MDA locations), before and after the first round of MDA—Angumu health zone, Ituri province, Democratic Republic of Congo, March 2021

	Non MDA locations			MDA locations		
	Deaths/10,000/day	95%CI	Design effect	Deaths/10,000/day	95%CI	Design effect
All causes						
All ages						
Before MDA (1)	0.67	[0.46–0.88]	2.55	0.54	[0.35–0.72]	1.54
Before MDA (2)	1.07	[0.84–1.30]	2.08	0.99	[0.68–1.29]	2.49
After MDA	0.92	[0.78–1.06]	0.89	0.80	[0.51–1.08]	2.58
Children under 5 years						
Before MDA (1)	2.78	[1.79–3.77]	2.07	1.63	[0.89–2.36]	1.27
Before MDA (2)	2.74	[2.08–3.4]	1.09	2.32	[1.48–3.16]	1.36
After MDA	2.67	[1.84–3.5]	1.76	1.10	[0.5–1.71]	1.55
Malaria-specific mortality						
All ages						
Before MDA (1)	0.36	[0.21–0.51]	2.47	0.33	[0.18–0.47]	1.50
Before MDA (2)	0.56	[0.43–0.69]	1.33	0.44	[0.25–0.63]	2.15
After MDA	0.39	[0.27–0.5]	1.38	0.28	[0.14–0.42]	1.85
Children under 5 years						
Before MDA (1)	2.07	[1.31–2.82]	1.63	1.47	[0.8–2.15]	1.17
Before MDA (2)	2.05	[1.42–2.67]	1.31	1.72	[0.89–2.55]	1.79
After MDA	1.71	[1.16–2.26]	1.23	0.78	[0.21–1.36]	1.96

*95% CI* 95% confidence interval, *MDA* Mass drug administration, Before MDA (1): October 2019-March 2020; Before MDA (2): April-September 2020; After MDA: October 2020-March 2021

The U5MR was above emergency threshold in “non-MDA” and “MDA locations” before the MDA campaign (2.74 [2.08–3.40] and 2.32 [1.48–3.16] deaths/10 000 children/day, respectively). It remained above the emergency threshold in “non-MDA locations” after October (2.67 [1.84–3.50]) while it decreased sharply below the emergency threshold in “MDA locations” (1.10 [0.50–1.71]).

The malaria-specific mortality was very similar in the whole population in “non-MDA” and “MDA locations” before the MDA campaign (0.56 [0.43–0.69] and 0.44 [0.25–0.63] malaria-related deaths/10 000 population/day, respectively) and remained very similar after the MDA campaign started (0.39 [0.27–0.50] and 0.28 [0.14–0.42], respectively. Among children under 5 years of age, the malaria-specific mortality was very similar in the “non-MDA” and “MDA locations” before the MDA campaign (2.05 [1.42–2.67] and 1.72 [0.89–2.55]] malaria-related deaths/10 000 children/day respectively). It decreased in both strata, but slightly more in “MDA locations” (0.78 [0.21–1.36]) than in “non-MDA locations” (1.71 [1.16–2.26]).

The survival curves for “MDA” and “non-MDA locations”, drawn through the weighted Kaplan–Meier (Additional file 3: Figure S2) and compared with the log-rank test, were not different “before” the MDA, neither for all-cause mortality not for malaria-specific mortality. However, for the period “after MDA” (from October 1^st^ to study date), if the survival curves were not different for all-cause mortality among whole population (p = 0.510), they were significantly different among children under 5 years of age (p = 0.003). Malaria-specific survival curves were significantly different among children under 5 (p = 0.007) and tended to be different for the whole population (p = 0.063).

Finally, based on the results of the Poisson regression, there was no difference in the mortality indicators (CMR, U5MR and malaria-specific mortality) between “MDA” and “non-MDA locations” before the MDA campaign (Table 3). After the MDA campaign started, there was still no difference in the all-cause mortality. However, the U5MR was 2.17 [1.36–3.49] higher in “non-MDA locations” than in “MDA locations”, malaria-specific mortality was 2.60 [1.56–4.33] higher in “non-MDA locations” among children under 5 years of age and 1.78 [1.12–2.82] higher in “non-MDA locations” among whole population.

**Table 3:** Adjusted mortality rate ratio in non-MDA locations compared to MDA locations, “Before” and “After” the MDA campaign– Angumu health zone, Ituri, DRC, 2021

	« Before» (01/10/2019–01/10/2020)		« After» (01/10/2020–26/03/2021)	
	aMRR	[95%CI]	aMRR	[95%CI]
Mortality (all causes)	1.09	[0.78–1.53]	1.32	[0.93–1.88]
Under 5 mortality (all causes)	1.15	[0.76–1.74]	2.17	[1.36–3.49]
Malaria-specific mortality	1.12	[0.74–1.70]	1.78	[1.12–2.82]
Malaria-specific mortality in < 5 years	1.15	[0.75–1.78]	2.60	[1.56–4.33]

*aMRR* mortality rate ratio, adjusted on age, sex, status (autochthonous/displaced) and mortality “before”, *95% CI* 95% confidence interval, *MDA* Mass drug administration

Reference = MDA locations (locations where three rounds of MDA have been implemented between September 2020 and January 2021)

Malaria or anaemia was the main cause of death reported by the heads of households, both in children under 5 years of age (66.0% in villages and 72.3% in IDP camps) and in the rest of the population (31.4% in villages and 31.7% in IDP camps) (Table 4).

**Table 4:** Details of the deaths that occured between September 2019 and March 2021 in Angumu health zone, Ituri province, DRC

	Village		IDP camps	
	< 5 years	≥ 5 years	< 5 years	≥ 5 years
Total number of deaths	191 (100%)	210 (100%)	83 (100%)	161 (100%)
By status				
Autochthonous	137 (71.7%)	150 (71.4%)	2 (2.4%)	4 (2.5%)
Displaced	54 (28.3%)	60 (28.6%)	81 (97.6%)	157 (97.5%)
By age				
< 1 month	38 (19.9%)		8 (9.6%)	
< 1 an	105 (55%)		29 (34.9%)	
5–9 years		26 (12.4%)		18 (11.2%)
By cause				
Malaria or anemia	126 (66.0%)	66 (31.4%)	60 (72.3%)	51 (31.7%)
Respiratory issue	4 (2.1%)	20 (9.5%)	3 (3.6%)	16 (9.9%)
Diarrhea/Vomiting	6 (3.1%)	7 (3.3%)	7 (8.4%)	14 (8.7%)
Accident	2 (1%)	15 (7.1%)	1 (1.2%)	12 (7.5%)
Violence	0 (0%)	4 (1.9%)	1 (1.2%)	18 (11.2%)
Delivery complication	20 (10.5%)	2 (1%)	1 (1.2%)	4 (2.5%)
Other	31 (16.2%)	91 (43.4%)	7 (8.4%)	45 (27.9%)
Unknown	2 (1%)	5 (2.4%)	3 (3.6%)	1 (0.6%)
Access to health care before death				
Hospital	74 (38.7%)	98 (46.7%)	28 (33.7%)	64 (39.8%)
Health center	92 (48.2%)	88 (41.9%)	61 (73.5%)	77 (47.8%)
Health post	0 (0%)	0 (0%)	0 (0%)	0 (0%)
Community care sites (IDP camps)	2 (1%)	3 (1.4%)	16 (19.3%)	11 (6.8%)
None	0 (0%)	0 (0%)	0 (0%)	0 (0%)

### Morbidity

In the 2 weeks prior to the survey (2.5 months after the last round of MDA), 49.8% [47–52] (deff = 5.9) of the population had been sick, and 57.4% [54–61] (deff = 2.4) of the children under 5 years of age. All morbidity indicators were significantly higher in “non-MDA locations” than in “MDA locations” (Table 5). The all-age morbidity was 1.89 [1.53–2.22] times higher in “non-MDA locations” (marginal morbidity equals to 52.1% [47.2–57.1] in “non-MDA locations” and 27.6% [22.4–32.7] in “MDA locations”. The morbidity among children under 5 years of age was 1.45 [1.27–1.66] times higher in “non-MDA locations”. Finally, the malaria-specific morbidity was 1.93 [1.59–2.34] and 2.13 [1.67–2.72] times higher in “non-MDA locations” among children under 5 years of age and whole population, respectively.

**Table 5:** Marginal morbidity and adjusted risk ratio (aRR) of being sick 2 weeks prior to survey, depending on MDA status, Angumu health zone, Ituri province, DRC, March 2021

	Non-MDA locations			MDA locations			aRR*	[95%CI]
	n	Marginal morbidity* (%)	[95%CI]	n	Marginal morbidity* (%)	[95%CI]		
All causes morbidity								
All ages	4854	52.1	[47.2–57.1]	1758	27.6	[22.4–32.7]	1.89	[1.53–2.22]
Children < 5 years	1076	70.0	[65.9–74.2]	522	48.3	[42.4–54.1]	1.45	[1.27–1.66]
Malaria-specific morbidity								
All ages	2646	29.3	[26.5–32.1]	849	13.7	[10.5–17.0]	2.13	[1.67–2.72]
Children < 5 years	684	44.0	[40.0–48.0]	239	22.8	[18.9–26.6]	1.93	[1.59–2.34]

*aRR* adjusted risk ratio * adjusted on age, sex and status (autochthonous/displaced), *95% CI* 95% confidence interval, *MDA* Mass drug administration

Reference = MDA locations = locations where three rounds of MDA have been implemented between September 2020 and January 2021

Non-MDA locations = locations where MDA has not been implemented in 2020

The main reported cause of morbidity was malaria, which was reported as the cause of morbidity for half of the sick people. For children under 5 years, this proportion was slightly higher in non-MDA locations (62% and 65% in villages and IDP camps, respectively) than in MDA locations (46% in villages and camps).

## Discussion

To our knowledge, this was the survey assessing the short-term effect of an MDA campaign for malaria conducted in high malaria prevalence area outside of an Ebola context, in a context of complex emergency and in the midst of Covid-19 pandemic.

### Key results

The survey carried out in March 2021 in Angumu health zone shows very high coverage and adherence of the population to the MDA carried out at the end of 2020 in four HAs. The door-to-door strategy, even though heavy to implement, most probably contributed to this success. It also shows a very strong reduction in U5MR and malaria-specific mortality (all age and  < 5 years), in HAs that have benefited from the MDA campaign, compared to non-targeted HAs, as well as in the morbidity (all age and  < 5 years, all cause and malaria-specific) more than 2 months after the end of the 3rd round of MDA.

### Comparison with other MDA surveys

The composition of the population studied in March 2021 seems consistent with previous surveys conducted in the health zone [1, 2]: the age pyramids or the proportion of autochthonous people and IDPs in villages and IDP sites were similar. The mortality and morbidity indicators estimated in non-MDA locations or prior to MDA were also in similar range than in previous surveys conducted in this health zone.

To our knowledge, there are no similar survey assessing the impact of MDA in a complex context with high malaria prevalence outside of Ebola context.

In 2014, during the Ebola outbreak, two rounds of MDA with ASAQ were carried out in four neighbourhoods of Monrovia (Liberia) and the districts hardest hit by the outbreak in Sierra Leone. Routine data were analysed to assess the short-term effect of this intervention. In Sierra Leone [9], several key malaria indicators decreased significantly on week 1 post-MDA and remained low on weeks 2 and 3: the number of suspected cases tested with rapid diagnostic test (RDT) decreased significantly by 43% (95% CI 38–48%); the RDT positive cases decreased significantly by 47% (41–52%); the total malaria (clinical + confirmed) cases decreased significantly by 45% (39–52%); the proportion of confirmed malaria cases (out of all-outpatients) fell by 33% (29–38%). On the contrary, the non-malaria outpatient cases either remained unchanged or fluctuated insignificantly. In Liberia [10], the incidence of self-reported fever decreased from 4.2% (52/1229) in the month prior to first round to 1.5% (18/1229) after (p < 0.001) and the decrease was larger among household members completing ASAQ (risk difference = 4.9%) compared to those not initiating ASAQ (Risk difference = 0.6%) (p < 0.001).

There have been other studies conducted in pre-elimination context, with mixed results. In Zanzibar [11], where a cluster randomized controlled trial of two rounds of MDA was conducted, no difference in cumulative malaria case incidence was observed between the control and intervention arms 6 months post-MDA (4.2 and 3.9 per 1000 population; p = 0.94). Neither was there a difference in PCR-determined parasite prevalence 3 months post-MDA (1.4% and 1.7%; OR = 1.0, p = 0.94), although having received at least the first MDA was associated with reduced odds of malaria infection (aOR = 0.35; p = 0.02). Conversely, in Zambia [12] where four rounds of MDA or focal MDA using dihydroartemisinin–piperaquine (DHAp) were conducted, infection incidence declined dramatically across all study arms during the period of study, and MDA was associated with reduced risk of first infection (hazards ratio: 0.36; 95% CI: 0.16–0.80) and cumulative infection incidence during the first rainy season (incidence rate ratio: 0.34; 95% CI 0.12–0.95). However, no significant effect was found for focal MDA or for either arm over the full study period.

### Limitations

Assessing the impact of such MDA campaign in complex emergency settings, some usual limitations of any population-based survey should be considered. Some bias may a priori be similar in MDA and non-MDA locations and therefore not affect the results. As for any retrospective survey relying on respondent recall, a potential memory bias could have happened. The main indicators were calculated based on what the head of household reported. No death certificate was consulted, no clinical examination by medical staff or confirmatory biological test was carried out during the investigation. The absolute malaria mortality and morbidity values should therefore be taken with caution. However, these biases would be similar in MDA and non-MDA locations, not affecting much the comparisons.

The data was analysed with “health area approach”, i.e. by classifying individuals according to their health area of residence and not according to the actual receipt of the drugs. Some people living in MDA locations may not have received the drugs, but the estimated coverage for the three rounds is very high. Conversely, some people living in non-MDA locations may have received the drugs after travelling to the villages where the activity took place. In both cases, this bias would lead to underestimating the observable effect when comparing mortality and morbidity in MDA and non-MDA locations.

What was called the “After” period is a mix of “during the three rounds of MDA” and “after the three rounds of MDA”. To differentiate the “during” effect from the “after” effect, a sub-analysis of mortality was conducted by starting the “after” period successively on January 25th (4 weeks after the average date of the 3rd round of MDA) and on February 8th (6 weeks after the average date of the 3rd round of MDA). No significant effect on mortality could be demonstrated for these two sub-periods “after the three rounds” through Kaplan–Meier estimates and Poisson regression. However, the recall period for this sub-analysis was very small, and the power insufficient to demonstrate a difference if there was one.

The departure date for people who left the household during the recall period was not collected. This could have introduced a bias in the estimation of mortality rates, in particular an under-estimation of the mortality rate in the MDA locations if the proportion of people who left there had been high. However, the proportion of people who left was low, and even lower in areas where MDA took place.

## Conclusions

The MDA campaign organized in Angumu health zone from September 2020 to January 2021 was a success in several aspects. It appears that implementing MDA was feasible, well accepted, and impactful in complex emergency context in a malaria holoendemic area. Indeed, the coverage of the different rounds and adherence to the treatments taken in the days following the distribution was very high, both in villages and IDP camps. Mortality in children under 5 years of age during and just after MDA was halved in HAs that benefited from MDA. Morbidity, and in particular malaria morbidity, was also divided by two in MDA locations 2.5 months after the last round of MDA.

These findings suggest MDA for malaria can be an important tool for rapidly reducing malaria morbidity and mortality in emergency contexts with high malaria burden. As this survey was conducted only few weeks after the MDA campaign, further evidence is needed to determine the duration of positive effect and the optimal timing and spacing of MDA rounds. Additional surveys assessing the best combination package of malaria control interventions would also be very useful, including the newly approved malaria vaccines.

## Supplementary Information

**Additional file 1. **Age pyramid of surveyed population in Angumu health zone, March 2021.

**Additional file 2. **Mortality rates in Angumu health zone, March 2021.

**Additional file 3: Figure S3.** Survival curves based on weighted Kaplan–Meier estimates in locations where MDA has been implemented (MDA locations, in red) and where it has not (non MDA locations, in blue), before and after the first round of MDA (October, 1 2020) in Angumu health zone, Ituri province, DRC, March 2021.

## Data Availability

The datasets used and/or analysed during the current study are available from the corresponding author on reasonable request.

## Abbreviations

aMRR: Adjusted mortality rate ratio
aRR: Adjusted risk ratio
ASAQ: Artesunate amodiaquine
CI: Confidence interval
CMR: Crude mortality rate
DHAp: Dihydroartemisinin–piperaquine
DRC: Democratic Republic of Congo
HA: Health area
IDP: Internally displaced people
MDA: Mass drug administration
MoH: Ministry of health
MSF: Médecins sans Frontières
U5MR: Under-five mortality rate
