# Supplementary material for: Effect of large-scale mass drug administration for malaria on mortality and morbidity in Angumu health zone, Ituri, Democratic Republic of Congo
Source: Malar J. 2023 Feb 6;22:44. doi: 10.1186/s12936-023-04469-7 (PMC9901819; doi:10.1186/s12936-023-04469-7)
Supplement: Supplementary file 1 — Additional file 1. Age pyramid of surveyed population in Angumu health zone, March 2021. [file 12936_2023_4469_MOESM1_ESM.pdf]

## Additional file S1 : Age pyramid of surveyed population in Angumu health zone, March 2021.

*Figure S1a : Age pyramid of surveyed population in the villages and IDP camps – Survey March 2021, Angumu health zone, Ituri province, Democratic Republic of Congo*

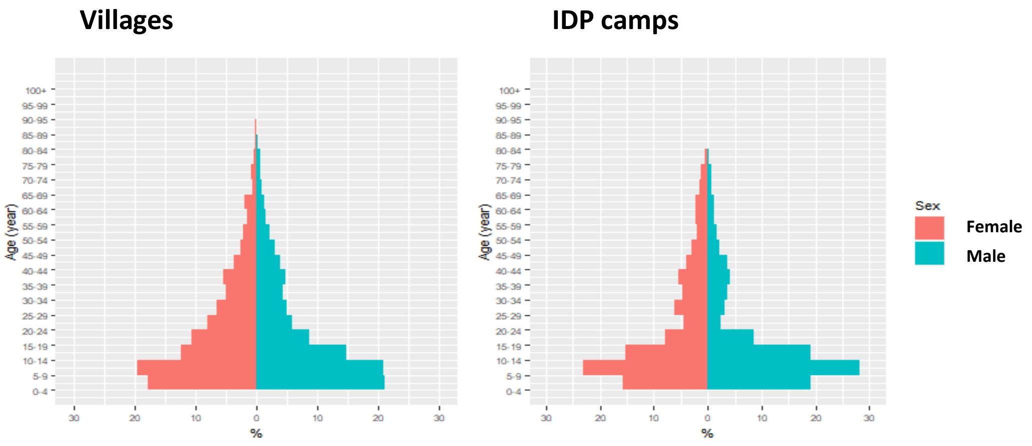


*Figure S1b : Age pyramid of surveyed population in the villages and IDP camps, according to whether they received MDA (on the left) or not (on the right) – Survey March 2021, Angumu health zone, Ituri province, Democratic Republic of Congo*

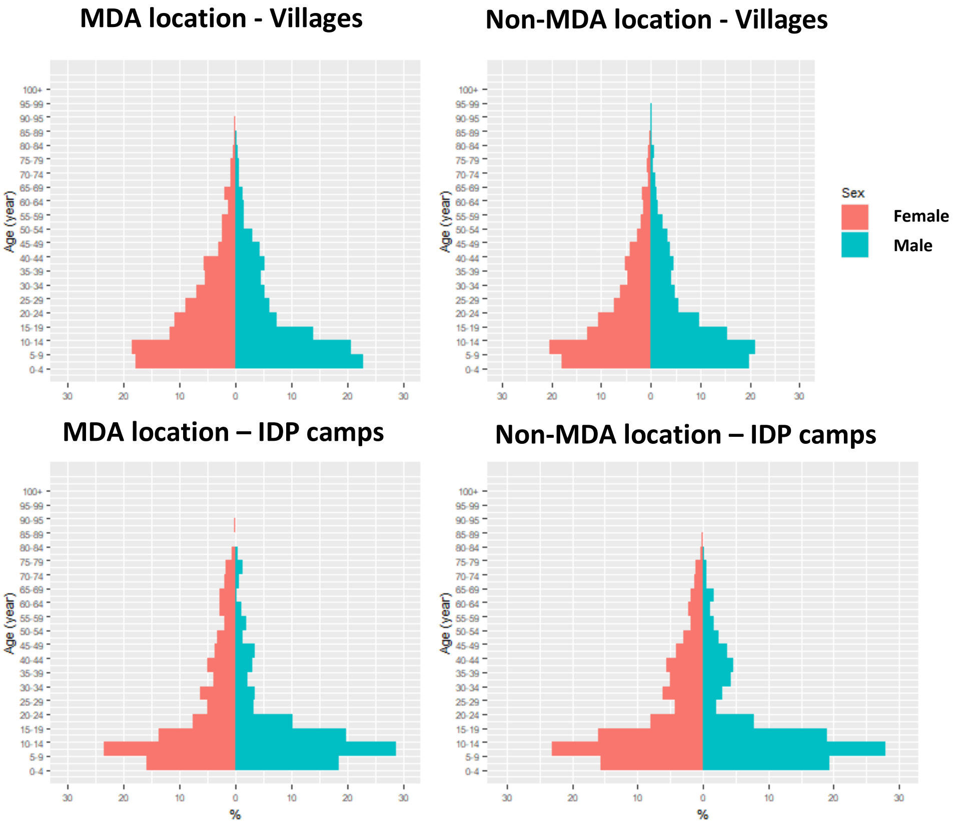


*IDP* Internally displaced people; *MDA* Mass drug administration
