# Supplementary material for: Effect of large-scale mass drug administration for malaria on mortality and morbidity in Angumu health zone, Ituri, Democratic Republic of Congo
Source: Malar J. 2023 Feb 6;22:44. doi: 10.1186/s12936-023-04469-7 (PMC9901819; doi:10.1186/s12936-023-04469-7)
Supplement: Supplementary file 2 — Additional file 2. Mortality rates in Angumu health zone, March 2021. [file 12936_2023_4469_MOESM2_ESM.pdf]

Additional file S2: Mortality rates in Angumu health zone, March 2021.

Table S1: Mortality rates in health areas where MDA has not been implemented and others where it has been implemented, for the whole population and children under 5 years of age – Angumu health zone, Ituri province, DRC – Survey March 2021.

	All ages						Children < 5 years of age					
	No MDA			MDA			No MDA			MDA		
	CMR	95%CI	Design effect	CMR	95%CI	Design effect	U5MR	95%CI	Design effect	U5MR	95%CI	Design effect
**Villages**												
**Before MDA (1)**	0.6	[0.44-0.76]	1.04	0.54	[0.37-0.72]	1.15	2.07	[1.23-2.90]	1.51	1.25	[0.69-1.82]	1.03
**Before MDA (2)**	0.94	[0.71-1.17]	1.45	0.79	[0.54-1.04]	1.64	2.43	[1.5-3.36]	1.63	2.25	[1.37-3.12]	1.36
**After MDA**	**1.01**	**[0.77-1.26]**	**1.38**	**0.80**	**[0.51-1.08]**	**1.99**	**3.09**	**[1.97-4.20]**	**1.65**	**1.01**	**[0.46-1.56]**	**1.1**
**IDP camps**												
**Before MDA (1)**	0.54	[0.32-0.75]	1.27	0.38	[0.04-0.73]	1.28	1.35	[0.56-2.14]	1.26	0.64	[0.00-1.52]	0.96
**Before MDA (2)**	1.04	[0.74-1.34]	1.4	1.22	[0.68-1.77]	1.31	2.25	[1.17-3.32]	1.45	2.42	[0.37-4.47]	1.69
**After MDA**	**0.81**	**[0.56-1.05]**	**1.15**	**0.72**	**[0.38-1.06]**	**0.95**	**2.00**	**[1.03-2.97]**	**1.18**	**0.58**	**[0-1.37]**	**0.99**

IDP=Internally Displaced People; MDA=Mass Drug Distribution; CMR=Crude mortality rate; U5MR=Under 5 mortality rate; CI=Confidence interval; Before MDA (1)=October 2019-March 2020; Before MDA (2)=April-September 2020; After MDA=October 2020-March 2021

Table S2: Malaria-specific mortality rates in health areas where MDA has not been implemented and others where it has been implemented, for the whole population and children~~ n~~ under 5 years of age – Angumu health zone, Ituri province, DRC – Survey March 2021.

	All ages						Children < 5 years of age					
	No MDA			MDA			No MDA			MDA		
	MMR	~~IC~~ 95%CI	~~Effet grappe~~Design effect	MMR	~~IC~~ 95%CI	Design effect~~Effet grappe~~	U5MMR	~~IC~~ 95%CI	Design effect~~Effet grappe~~	U5MMR	~~IC~~ 95%CI	Design effect ~~Effet grappe~~
**Villages**												
**Before MDA~~: Oct 2019-March 2020~~ (1)**	0.33	[0.20-0.45]	1.14	0.30	[0.16-0.44]	1.29	1.44	[0.78-2.1]	1.36	1.02	[0.51-1.53]	0.99
**Before MDA~~: April 2020- Sept 2020~~ (2)**	0.50	[0.35-0.66]	1.20	0.42	[0.23-0.61]	1.74	1.70	[0.93-2.47]	1.6	1.52	[0.79-2.26]	1.39
**After MDA~~: Oct 2020-Machs 2021~~**	**0.49**	**[0.33-0.65]**	**1.30**	**0.30**	**[0.16-0.45]**	**1.39**	**1.85**	**[1.09-2.6]**	**1.27**	**0.61**	**[0.16-1.07]**	**1.25**
**Whole recall period: Sept 2019, March 2021**	0.42	[0.33-0.52]	1.63	0.33	[0.23-0.43]	1.95	1.79	[1.29-2.29]	1.75	1.11	[0.75-1.47]	1.28
**IDP camps**												
**Before MDA~~: Oct 2019-March 2020~~ (1)**	0.47	[0.26-0.69]	1.43	0.26	[0.01-0.51]	0.97	1.64	[0.71-2.57]	1.45	0.65	[-0.23-1.54]	0.96
**Before MDA~~: April 2020- Sept 2020~~ (2)**	0.63	[0.39-0.87]	1.42	0.45	[0.14-0.76]	1.12	1.96	[0.96-2.97]	1.45	1.14	[-0.16-2.43]	1.37
**After MDA~~: Oct 2020-Machs 2021~~**	**0.46**	**[0.28-0.65]**	**1.12**	**0.23**	**[0.03-0.42]**	**0.99**	**1.69**	**[0.81-2.56]**	**1.13**	**0.29**	**[-0.27-0.85]**	**0.99**
**Whole recall period: Sept 2019, March 2021**	0.50	[0.38-0.62]	1.41	0.33	[0.19-0.47]	0.94	1.88	[1.29-2.47]	1.38	0.77	[0.18-1.35]	1.08

MDA=Mass Drug Distribution; MMR=Malaria-specific mortality rate; U5MMR=Under 5 malaria-specific mortality rate; CI=Confidence interval; Before MDA (1)=October 2019-March 2020; Before MDA (2)=April-September 2020; After MDA=October 2020-March 2021
