# Supplementary material for: Effect of large-scale mass drug administration for malaria on mortality and morbidity in Angumu health zone, Ituri, Democratic Republic of Congo
Source: Malar J. 2023 Feb 6;22:44. doi: 10.1186/s12936-023-04469-7 (PMC9901819; doi:10.1186/s12936-023-04469-7)
Supplement: Supplementary file 3 — Additional file 3: Figure S3. Survival curves based on weighted Kaplan–Meier estimates in locations where MDA has been implemented (MDA locations, in red) and where it has not (non MDA locations, in blue), before and after the first round of MDA (October, 1 2020) in Angumu health zone, Ituri province, DRC, March 2021. [file 12936_2023_4469_MOESM3_ESM.pdf]

Figure S3 : Survival curves based on weighted Kaplan-Meier estimates in locations where MDA has been implemented (MDA locations, in green) and where it has not (non MDA locations, in orange), before and after the first round of MDA (October, 1 2020) in Angumu health zone, Ituri province, DRC, March 2021.

a) All causes mortality

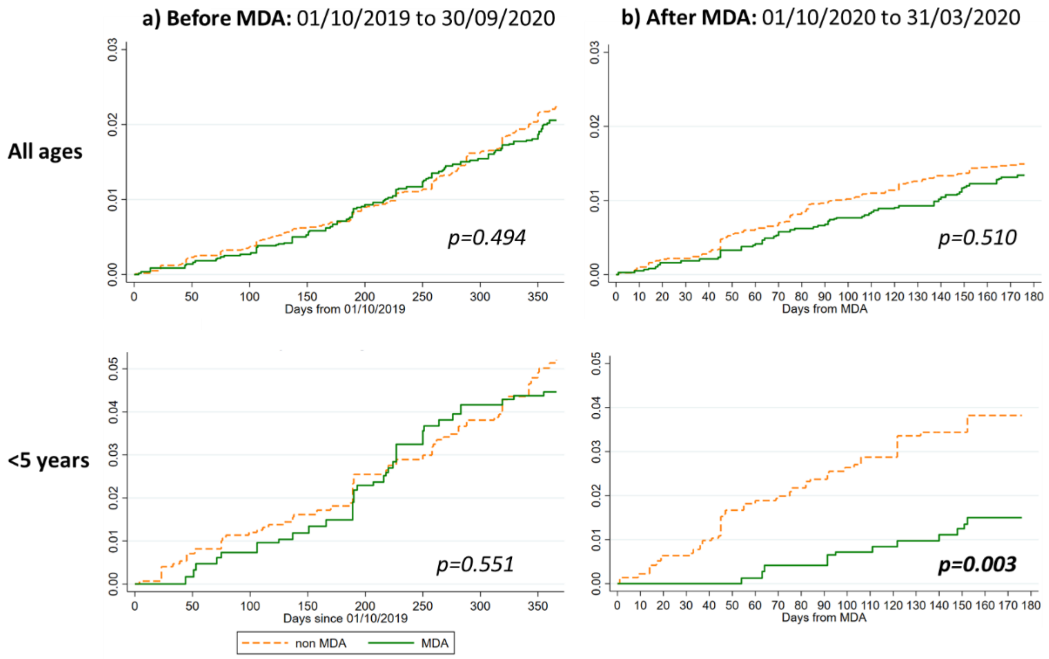

b) Malaria-specific mortality

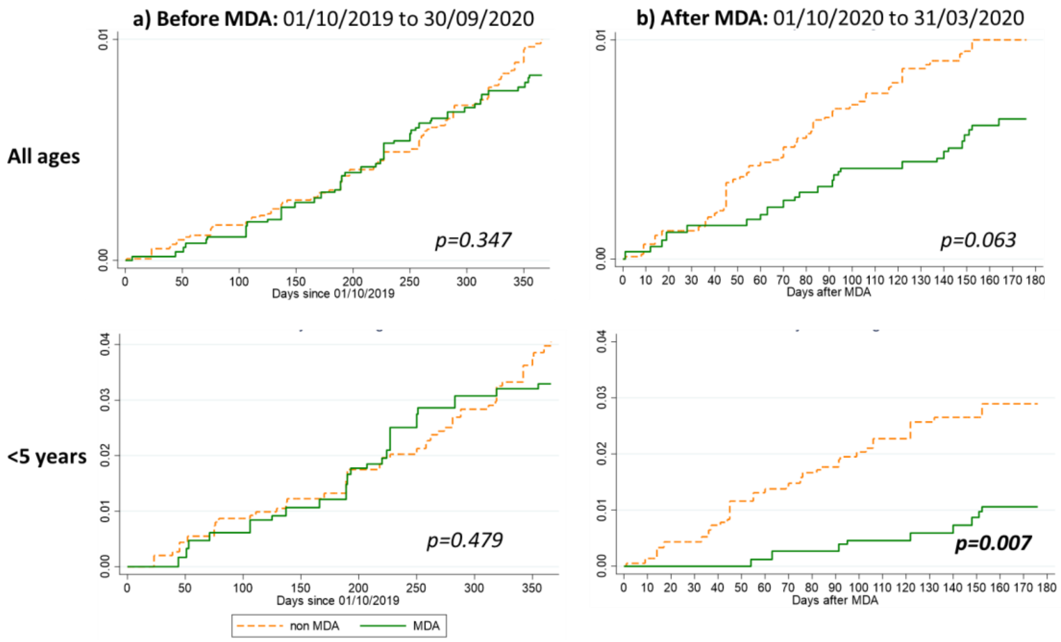

P-value are based on log-rank tests and are considered significant (bold) if $p<0.05$.
